# HSC engraftment is enhanced by combining mobilization with anti-C-Kit and Anti-CD47-based conditioning in hematopoietic transplant

**DOI:** 10.1016/j.ymthe.2025.07.012

**Published:** 2025-07-16

**Authors:** Isabel Ojeda-Perez, Omaira Alberquilla-Fernandez, Aida García-Torralba, Mercedes Lopez-Santalla, Rebeca Sánchez-Domínguez, Jose-Carlos Segovia

**Affiliations:** 1 Cell Technology Division, Centro de Investigaciones Energéticas, Medioambientales y Tecnológicas (CIEMAT) and Centro de Investigación Biomédica en Red de Enfermedades Raras (CIBERER), Madrid, Spain; 2 Unidad Mixta de Terapias Avanzadas, Instituto de Investigación Sanitaria Fundación Jiménez Díaz (IIS-FJD, UAM), Madrid, Spain; 3 Division of Hematopoietic Innovative Therapies, Centro de Investigaciones Energéticas, Medioambientales y Tecnológicas (CIEMAT) and Centro de Investigación Biomédica en Red de Enfermedades Raras (CIBERER), Madrid, Spain

**Keywords:** Non-genotoxic, conditioning, monoclonal antibody, HSC mobilizers, Rag-2 deficiency, immunodeficiency, pyruvate kinase deficiency, anemia

## Abstract

A significant limitation of hematopoietic stem cell transplantation (HSCT) that reduces its application across more disease areas and more geographically diverse populations is the toxicity from chemotherapy-based conditioning. A potential solution is to replace chemotherapy with monoclonal antibodies, but the replacement must result in therapeutically relevant levels of engraftment. In some cases, this level of engraftment can be quite low (<10%) but in other situations must be significantly higher. Naked monoclonal antibody therapy (without using a potentially toxic drug conjugate) alone has been inconsistent in generating high levels of engraftment. Agents that mobilize hematopoietic stem and progenitor cells (HSPCs) out of the bone marrow niche are safely used as a method to harvest HSPCs as a source of cells for HSCT. We hypothesized that mobilization might sensitize HSPCs to monoclonal antibody depletion to facilitate high levels of donor cell engraftment. We provide evidence to support this hypothesis by showing in different mouse models of HSCT that mobilization consistently, safely, and reproducibly generates higher levels of engraftment when combined with a specific monoclonal antibody conditioning cocktail compared with monoclonal antibody therapy alone. This combination therapy is a promising approach to allowing HSCT to be applied to more diseases and broader populations than current chemotherapy-based conditioning permits.

## Graphical abstract

**Figure:**
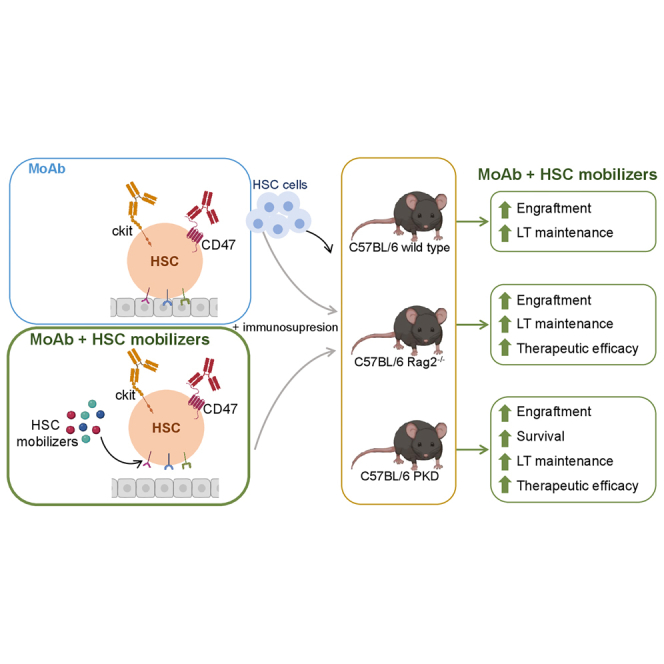

## Teaser

The authors demonstrate that combining hematopoietic stem cell (HSC)-specific antibodies with HSC mobilizing agents enables effective, non-genotoxic conditioning for HSC transplantation. This strategy achieves long-term, multilineage engraftment, therapeutically efficient, across mouse models of human diseases and offers a promising, clinically translatable alternative to genotoxic chemotherapy-based conditioning.

## Introduction

Hematopoietic stem cell transplantation (HSCT) is a critical therapeutic approach for treating cancers and blood disorders that replaces patients’ damaged blood-forming cells with healthy ones capable of generating new, functional blood cells. Over 1.5 million HSCT procedures have been performed over the past 60 years.^1^ Achieving successful engraftment rates of healthy cells in most patients requires host hematopoietic stem cell (HSC) ablation to eliminate diseased cells, create space for exogenous healthy stem cells, and suppress the immune system to prevent graft rejection. Traditional conditioning regimens that involve high-dose chemotherapy and/or total body irradiation are highly genotoxic and associated with significant severe adverse effects, including infections, graft failure, graft-vs.-host disease (GVHD), organ damage, secondary cancers, need for re-immunization, and infertility.^2^^,^^3^^,^^4^ In selected patient cohorts, durable engraftment can be achieved with reduced-intensity regimens, and in specific cases such as Fanconi anemia, sustained engraftment may occur even without conditioning.^5^ Nevertheless, to make transplantation more accessible, alternative non-genotoxic conditioning (NGC) strategies are required to reduce treatment-associated toxicity while ensuring therapeutic engraftment rates.

Recently, there has been a significant shift toward the adoption of NGC strategies to ensure effective conditioning with minimal toxicity. Emerging methods include reduced-intensity conditioning, immunomodulatory therapies, monoclonal antibody (MoAb)-based treatments, and HSC mobilization approaches. The U.S. Food and Drug Administration is currently evaluating more than 130 antibody therapeutics for a range of applications, including unconjugated antibodies, antibody-drug conjugates, and radiolabeled antibodies.^6^ Seven MoAbs targeting HSCs are currently in clinical trials as NGC alternatives,^7^^,^^8^^,^^9^^,^^10^^,^^11^^,^^12^ with at least five more in preclinical development.^13^^,^^14^^,^^15^^,^^16^^,^^17^ Research on MoAb targeting c-Kit shows promise in antibody-mediated HSC depletion.^1^^,^^16^^,^^18^^,^^19^^,^^20^^,^^21^^,^^22^^,^^23^ In studies conducted on mouse models, a combination of four antibodies (anti-c-Kit-ADC, anti-CD40L, anti-CD4, and anti-CD8)^24^ or six naked antibodies (anti-c-Kit, anti-CD47, anti-CD122, anti-CD40L, anti-CD4, and anti-CD8) was successfully used to facilitate engraftment of mismatched HSCs into immunocompetent recipient mice, which resulted in measurable donor chimerism. The last approach also induced tolerance to matched heart grafts while preserving immune responses against foreign tissues.^25^

Mobilization of hematopoietic stem and progenitor cells (HSPCs) from bone marrow (BM) to peripheral blood (PB) via drug induction has also emerged as a promising NGC strategy.^26^^,^^27^^,^^28^ Mobilization procedures use agents like granulocyte colony-stimulating factor (G-CSF),^18^ CXCR4 blockers^29^^,^^30^^,^^31^ (e.g., plerixafor [PX]), or very late antigen-4 (VLA-4) antagonists (i.e., BIO5192)^32^^,^^33^ to increase HSCs in PB by disrupting HSPC interactions with the BM niche.^34^

Despite these promising results, limitations persist in achieving therapeutic engraftment levels. To improve the efficacy of antibody-targeted treatments, we combined specific MoAb treatment with mobilization agents. By blocking HSC-niche interactions, we hypothesized that HSC mobilizers facilitate the migration of stem cells from the BM to PB allowing antibodies access to interact with HSC outside the protective BM niche. We demonstrated that this combined approach significantly enhances engraftment efficiency and improves survival rates. Moreover, we confirmed that this biomedical development enables an effective and long-term therapeutic efficacy in two mouse models of inherited hematopoietic diseases: Rag-2-deficiency (Rag2^−/−^),^35^ a life-threatening form of severe combined immunodeficiency (SCID) diseases characterized by severe defects in both T and B cell function that leads to extreme susceptibility to infections,^36^ and pyruvate kinase deficiency (PKD), a chronic hemolytic disorder that significantly impairs quality of life and life expectancy of the patients.^37^

## Results

### Hematopoietic conditioning with an anti-c-Kit and anti-CD47 combined with PX allows high autologous long-term multilineage engraftment in wild-type mice

To establish a non-genotoxic conditioning (NGC) protocol for high autologous engraftment levels, we combined anti-c-Kit and anti-CD47 antibodies MoAbs) with an immunosuppressive regimen^25^ and a hematopoietic stem cell mobilizer, specifically the CXCR-4/SDF1 antagonist (AMD3100 or plerixafor [PX]).

First, we studied the clearance kinetics of anti-c-Kit (ACK2 clone) and the mobilization dynamics induced by PX to optimize the timing of both antibody administration and HSPC transplantation (Figure S1). Analysis of anti-c-Kit clearance revealed that its serum levels declined sufficiently by day 4 post-injection. Based on this, we established day −5 as the optimal timing for anti-c-Kit administration to prevent any potential interference with HSPC engraftment (Figure S1A). To determine the optimal mobilization window, we injected PX into wild-type (WT) mice and collected blood samples every 30 min to measure the mobilization levels of different populations, focusing on c-Kit+ and lineage-negative (Lin^−^) Sca-1^+^c-Kit^+^ (LSK) cells. We observed that the peak of mobilization occurred at 1.5 hours after the injection. To synchronize this peak with anti-c-Kit administration, we determined that PX should be injected 1 hour before anti-c-Kit administration (Figure S1B).

**Figure 1:**
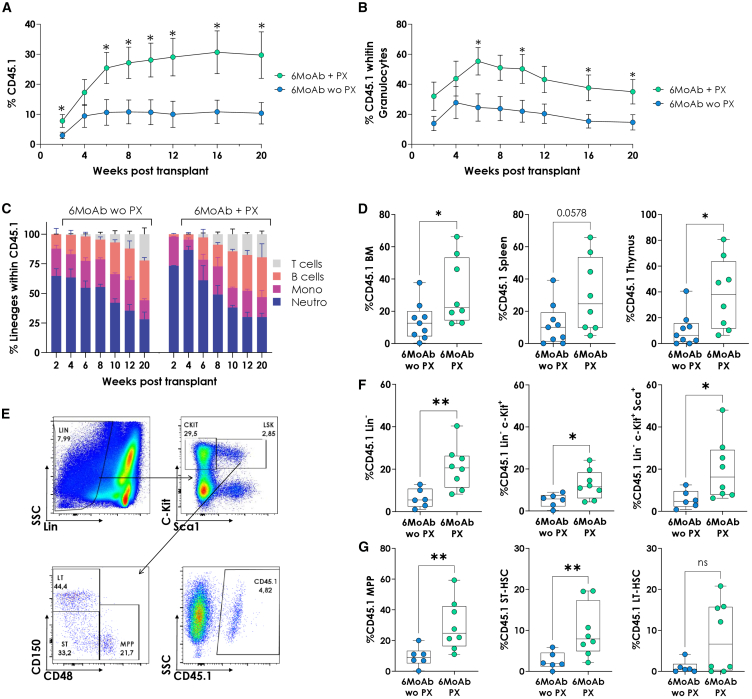
Combination of PX with 6-MoAb cocktail conditioning improves long-term multilineage hematopoietic reconstitution (A) Peripheral blood chimerism in CD45.2 wild-type mice transplanted with 15,000 CD45.1 LSK cells after MoAb conditioning with (PX) or without PX (woPX). (B) Granulocyte chimerism in CD45.2 wild-type mice transplanted with 15,000 CD45.1 LSK cells after MoAb conditioning with (PX) or without PX (woPX). (C) Kinetics of CD45.1 lineage reconstitution over time on primary recipients. T cells: CD3^+^; B cells: B220^+^; Mono: CD11b^+^Gr1^−^; Neutro: CD11b^+^ Gr1^+^, without (woPX) and with PX (PX). (D) Chimerism of CD45.1 cells after 6 months in the whole BM, spleen, and thymus. (E) Top: Representative dot plot showing gating strategy for the analysis of LSK and c-Kit progenitors. Bottom: Representative dot plot showing SLAM-based gating strategy for identification of more immature populations (MPP, CD48^+^CD150^−^; ST-HSC, CD48^−^CD150^−^; and LT-HSC, CD48^−^CD150^+^) and CD45.1 gating example. (F) Chimerism of CD45.1 cells after 6 months within Lin^−^, c-Kit^+^, and LSK^+^ progenitor populations. (G) Percentage of CD45.1 cells after 6 months within MPP, ST-HSC, and LT-HSC immature progenitors. (A) and (B) represent the mean and SEM from *n* = 8 without PX (wo PX) and *n*= 10 with PX (PX). Boxplots show the median, range, and quartiles of the data distribution. MoAb, monoclonal antibody.

To evaluate the engraftment of exogenous cells in NGC mice, we intravenously transplanted 15,000 healthy HSPCs (LSK^+^ cells: Lin^−^ Sca1^+^c-Kit^+^) from CD45.1 donors into CD45.2 recipients. Recipients were divided into two groups: antibody-based conditioning without PX (wo PX) and antibody-based conditioning with PX (PX), following the protocol represented in Figure S1C. Engraftment, determined by the presence of >1% donor cells in peripheral blood (PB) lineages, was periodically measured. The addition of PX significantly increased engraftment and increased total donor chimerism from ∼10% (wo PX) to ∼30% (PX) at 20 weeks post-transplantation (Figure 1A). Moreover, PX improved granulocyte-specific engraftment (∼14% wo PX vs. ∼35% PX, Figure 1B) and prevented engraftment failure (0% failure with PX vs. 20% without PX, Figure S1D). All hematopoietic lineages successfully engrafted in both groups (Figure 1C).

PX addition enhanced engraftment across all hematopoietic organs, including BM, spleen, and thymus (Figure 1D). To confirm HSC engraftment, we analyzed HSPC populations in the BM at 6 months post-transplantation (Figure 1E). PX-conditioned mice showed significantly higher engraftment in Lin^−^, c-Kit^+^, and LSK^+^ compartments (Figure 1F). In the more primitive HSC compartment, multipotent progenitor (MPP) and short-term HSC (ST-HSC) populations increased significantly, with a similar trend in long-term HSCs (LT-HSCs) (Figure 1G).

**Figure 2:**
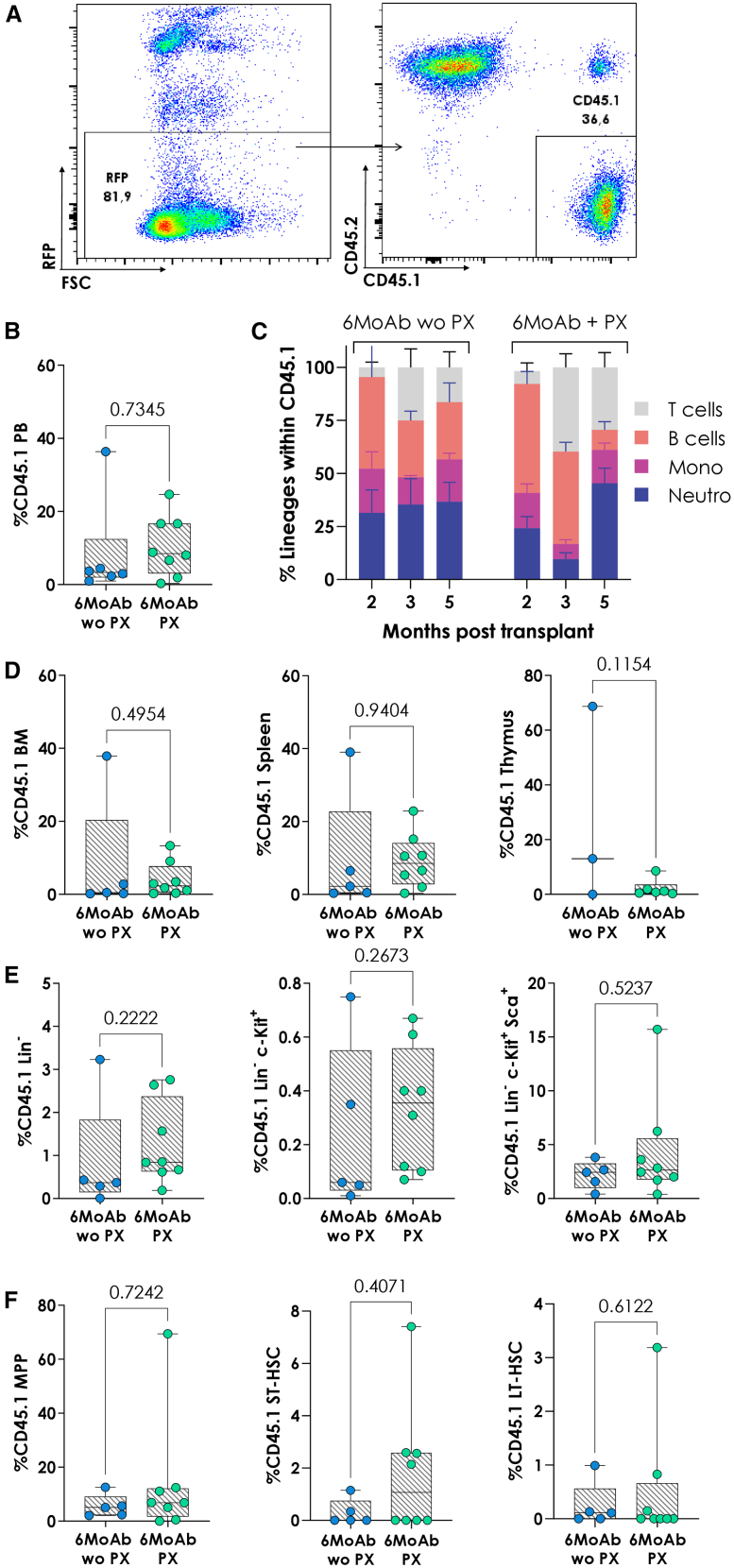
Combination of PX with 6-MoAb cocktail conditioning maintains engraftment in secondary recipients (A) Representative dot plot showing gating strategy for the analysis of engraftment in RFP mice. (B) Lineage reconstitution within the RFP^−^CD45.1^+^engraftment without (wo PX) and with PX (PX). Mean and SD are represented. T cells: CD3^+^; B cells: B220^+^; Mono: CD11b^+^Gr1^−^; Neutro: CD11b^+^ Gr1^+^. (C) Percentage of CD45.1 within Lin^−^, Lin^−^c-Kit^+^ and Lin^−^c-Kit^+^Sca-1^+^ (LSK) on secondary recipients. (D) Chimerism of CD45.1 cells after 5 months within MPP. (E) Chimerism of CD45.1 cells after 5 months within ST-HSC and LT-HSC immature progenitors. (F) Chimerism of CD45.1 cells after 5 months in BM, spleen, and thymus. The boxplots show the median, range, and quartiles of the data distribution. *n* = 5 without PX (wo PX) and *n* = 8 with PX (PX). MoAb, monoclonal antibody.

For long-term engraftment assessment, we performed secondary transplants using transgenic C57BL/6 mice expressing red fluorescent protein (RFP) (Figure 2A). Secondary recipient mice conditioned with lethal irradiation (9 Gy in two 4.5-Gy doses, 24 h apart) received 5–8 million BM cells from primary NGC mice. Engraftment was stable after 5 months in both groups. Both showed peripheral blood engraftment at this time point, with a slight increase observed in the group receiving antibodies plus mobilizers (Figure 2B) and multilineage reconstitution in secondary recipients (Figure 2C). No differences were observed in BM, spleen, or thymus between conditioning treatments regarding engraftment (Figure 2D). Moreover, engraftment was maintained across bone marrow progenitor populations, including LSK^+^ cells as well as more immature subsets such as MPPs, ST-HSCs, and LT-HSCs (Figures 2E and 2F). This indicates that our NGC protocol supports the engraftment of HSCs capable of repopulating secondary recipients.

Overall, combining MoAbs with PX significantly enhances HSC engraftment in an NGC setting. This promoted multilineage engraftment maintaining long-term hematopoietic reconstitution.

### Combination of an anti-c-Kit/anti-CD47 with PX enables high and therapeutic levels of engraftment in Rag2^−/−^ immunodeficient mice

**Figure 3:**
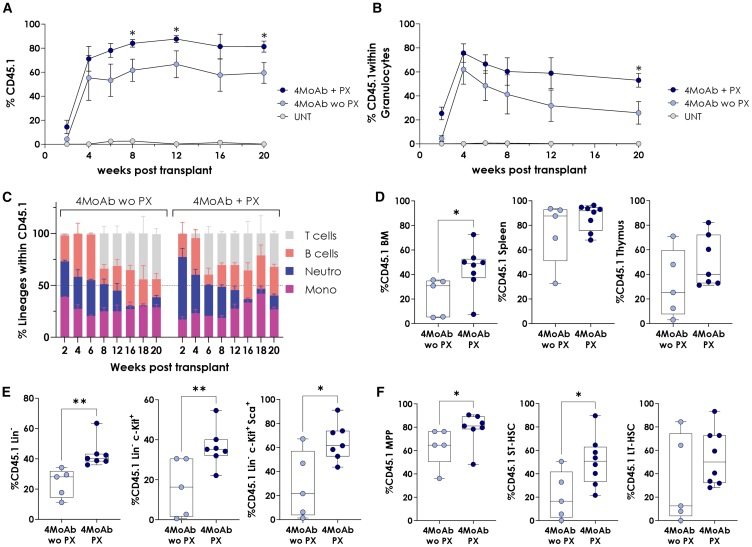
Combination of PX with 4-MoAb cocktail conditioning improves multilineage hematopoietic reconstitution in SCID mice (A) Peripheral blood chimerism in CD45.2 Rag2^−/−^ mice transplanted with 15,000 CD45.1 LSK cells following monoclonal antibody conditioning, with (PX) or without PX (woPX), as well as in untreated mice (UNT). (B) Granulocyte chimerism in CD45.2 Rag2^−/−^ mice transplanted with 15,000 CD45.1 LSK cells following monoclonal antibody (MoAb) conditioning, with (PX) or without PX (wo PX), as well as in untreated mice (UNT). UNT mice were not conditioned but were transplanted with 15,000 CD45.1 LSK cells. (C) Kinetics of CD45.1 lineage reconstitution over time on primary recipients. Lin T: CD3^+^; Lin B: B220^+^; Mono: CD11b^+^Gr1^−^; Gr: CD11b^+^ Gr1^+^. (D) Chimerism of CD45.1 cells in the whole BM, spleen, and thymus 5 months post-transplantation. (E) Chimerism of CD45.1 cells after 6 months within Lin^−^, c-Kit^+^, and LSK^+^ progenitor populations. (F) Percentage of CD45.1 cells within MPP, ST-HSC, and LT-HSC immature progenitors 6 months post-transplant. Mean and SEM are represented. The boxplots show the median, range, and quartiles of the data distribution. *n* = 5 without PX (wo PX) and *n* = 8 with PX (PX).

Once the advantage of the MoAb plus PX NGC protocol was demonstrated in WT mice, we aimed to test its therapeutic effect in animal models of monogenic diseases. First, we focused on Rag-2 deficiency (Rag2^−/−^), a type of severe combined immunodeficiency (SCID) characterized by defective lymphocyte development.^35^ These mice lack mature T and B cells but retain natural killer (NK) cells. Therefore, the conditioning regimen excluded anti-CD4 and anti-CD8 antibodies and consisted of a 4-MoAb cocktail: anti-c-Kit, anti-CD47, anti-CD40L, and anti-CD122. Fifteen thousand WT-CD45.1-LSK^+^ cells were infused into MoAb-conditioned Rag2^−/−^ mice, with and without PX. Consistent with the results in wild type, the combination of PX and MoAb significantly increased engraftment from ∼41% to ∼68% 5 months post-transplantation (Figure 3A). Additionally, this combination sustained granulocyte engraftment at ∼46%, whereas MoAb alone led to a sharp decline to ∼6% (Figure 3B). Engrafted CD45.1^+^ cells successfully differentiated into all PB lineages, with PX promoting uniform multilineage reconstitution and earlier T cell emergence (Figure 3C). PX also enhanced engraftment across BM, spleen, and thymus (Figure 3D). Immature HSC populations in BM were higher in PX-conditioned mice (Figures 3E and 3F).

**Figure 4:**
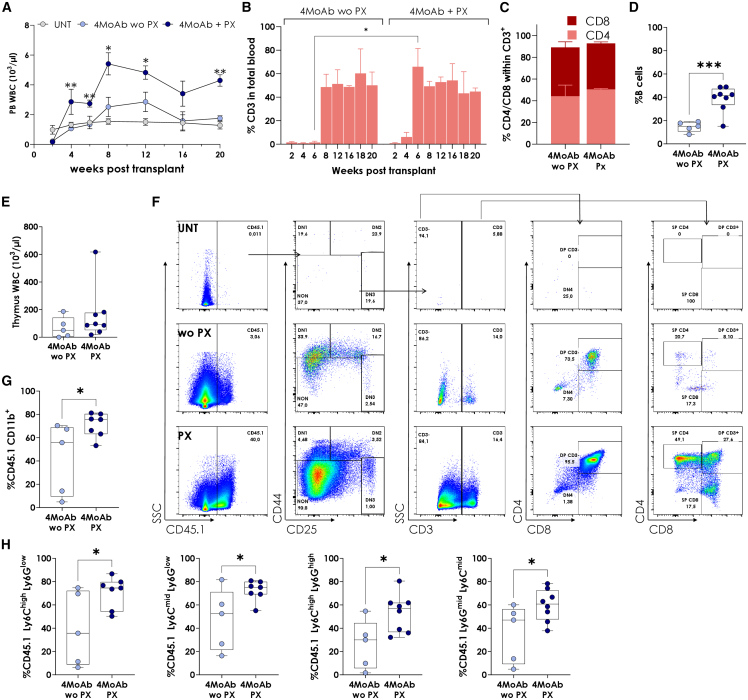
Impact of NGC treatment with and without PX in combination with the 4-MoAb cocktail on hematopoietic and thymic recovery (A) Kinetics of white blood cell counts (WBCs) in Rag2^−/−^ mice over time. (B) Reconstitution dynamics of T lymphocytes (CD3^+^) within PB without (wo PX) and with PX (PX). (C) CD4/CD8 ratio within the CD3^+^ population 5 months post-transplant. (D) Percentage of B lymphocytes within the total PB 5 months post-transplant. (E) Total WBC counts in the thymus 5 months post-transplant (F) Representative thymic development profiles within CD45.1^+^ cells for each treatment group. Top: mice transplanted with cells without conditioning (UNT). Middle: mice conditioned with MoAb alone (wo PX). Bottom: mice conditioned with the combination of MoAb and PX (PX). Thymic populations were gated as follows: DN1 (CD44^+^CD25^−^), DN2 (CD44^+^CD25^+^), DN3 (CD44^−^CD25^+^), DN4 (CD44^−^CD25^−^ CD3^−^CD8^+^), DP CD3^−^ (CD44^−^CD25^−^CD3^−^CD4^+^CD8^+^), DP CD3^+^ (CD44^−^CD25^−^ CD3^+^CD4^+^CD8^+^), SP CD4 (CD44^−^CD25^−^ CD3^+^CD4^+^) and SP CD8 (CD44^−^CD25^−^ CD3^+^CD8^+^). (G) Chimerism of CD45.1 cells within the myeloid (CD11b^+^) population. (H) Percentages of specific myeloid subpopulations in PB, including monocytes (CD11b^+^ Ly6C^high or mid^ Ly6G^low^), neutrophils (CD11b^+^ Ly6C^high^ Ly6G^high^), and myeloid progenitors (CD11b^+^ Ly6C^high^ Ly6G^mid^), in PX- and non-PX-treated mice. The boxplots show the median, range, and quartiles of the data distribution. *n* = 5 without PX (wo PX) and *n* = 8 with PX (PX). MoAb, monoclonal antibody.

To evaluate the recovery of functional immunocompetent hematopoiesis, we examined white blood cell (WBC) levels post-transplantation. PX-treated mice achieved significantly higher WBC levels and faster recovery compared with MoAb alone, where levels dropped to untreated control values by 5 months (Figure 4A). Engraftment dynamics showed that PX-treated mice exhibited CD3^+^ cells by 4 weeks post-transplantation, peaking at 6 weeks, while non-PX-treated mice showed delayed T cell recovery, peaking at 8 weeks (Figure 4B). Moreover, the absolute lymphocyte count was significantly higher in the plerixafor group compared with the antibody-only group, which showed values nearly indistinguishable from untreated controls (Figure S2A). By 5 months, both groups achieved balanced CD4^+^ and CD8^+^ T cell populations (Figure 4C). PX also increased in PB B cells 5 months post-transplantation (Figure 4D).

Thymic recovery was assessed by WBC quantification, which showed higher thymic cellularity in PX-treated mice (Figure 4E). Thymic architecture was more developed in PX-treated mice compared to non-PX-treated mice (data not shown). Thymic differentiation of engrafted CD45.1^+^ cells (Figure S2B)^38^^,^^39^ was markedly enhanced in PX-treated mice, with higher proportions of cells transitioning through advanced stages, including double-positive (DP) and single-positive (SP) CD4^+^ and CD8^+^ populations. In contrast, mice treated without PX showed delayed and reduced differentiation, with a significant accumulation of cells in earlier stages (Figures 4F, S2C, and S2D).

At 5 months post-transplantation, PX-treated mice showed significantly higher exogenous myeloid cell (CD11b^+^) contribution (∼52%) compared with non-PX-treated mice (∼10%) (Figure 4G). Similar trends were observed in monocytic (CD11b^+^ Ly6C^high or mid^ Ly6G^low^), neutrophilic (CD11b^+^ Ly6C^high^ Ly6G^high^), and immature populations (CD11b^+^ Ly6C^high^ Ly6G^mid^) (Figure 4H). Moreover, preliminary studies in secondary recipients indicated that engraftment levels were sustained for at least 3 months post-transplantation in MoAb + PX-treated mice, whereas MoAb-treated mice alone exhibited a drastic decline (data not shown).

Thus, the combination of MoAbs and PX enables high and multilineage exogenous engraftment in Rag2^−/−^ immunodeficient mice, which allows the recovery of a normal immunocompetent phenotype hematopoiesis.

### Addition of plerixafor enhances bone marrow depletion and protects from toxicity in a PKD model

**Figure 5:**
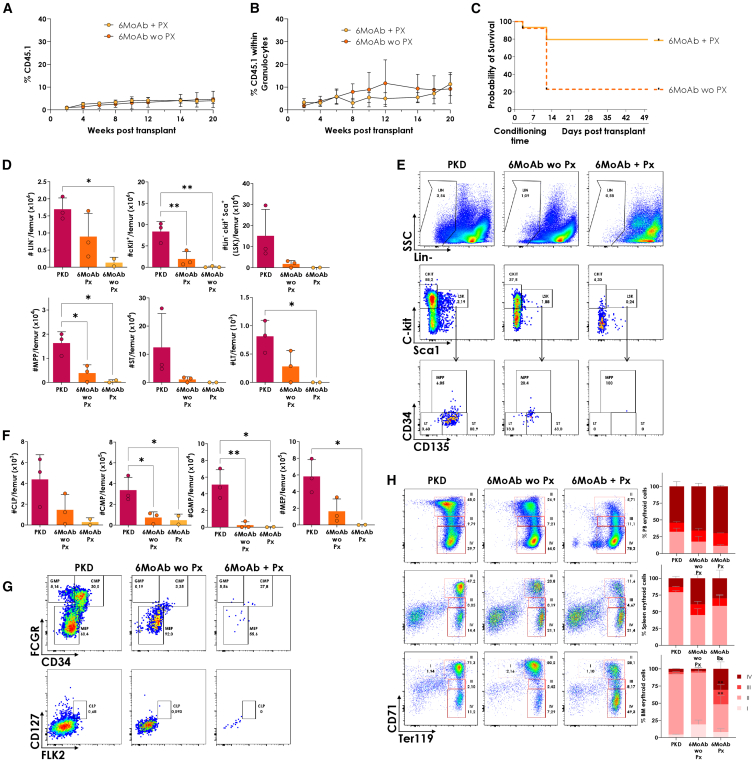
Hematopoietic conditioning with 6-MoAb cocktail plus PX allows multilineage hematopoietic reconstitution, increases survival rate and bone marrow depletion in an anemic mouse model of PKD (A) Engraftment percentages over time in PKD mice following the conditioning treatment, with (PX) or without PX (wo PX) administration and transplant with 15,000 LSK cells/mouse. (B) Granulocyte chimerism in CD45.2 PKD mice transplanted with 15,000 CD45.1 LSK cells following monoclonal antibody conditioning, with (PX) or without PX (wo PX). (C) Survival rates of PKD mice who underwent a conditioning treatment with the antibody cocktail, with or without PX. (D) Total number per femur of non-mature cells (Lin^−^), committed progenitors (Lin^−^c-kit^+^ and Lin^−^c-kit^+^ Sca-1^+^) and in immature phenotypes, such as MPP: LSK^+^ CD150^−^ CD48^+^; ST: LSK^+^ CD150^−^ CD48^−^; LT: LSK^+^ CD150^+^ CD48^−^ in the BM 5 days post c-Kit injection. (E) Representative dot plot of PKD and conditioned mice with or without PX, showing populations of Lin^−^, LSK, c-Kit^+^, and immature progenitor cells in the BM 5 days post c-Kit injection. (F) Total number per femur of lineage committed progenitors including CLP: LSK^+^ FLK2^+^ CD127^+^; CMP: LSK^+^CD34^+^FCRƔ^mid^; GMP: LSK^+^CD34^+^ FCRƔ^+^; MEP: LSK^+^ CD34^−^ FCRG^−^ 5 days post c-Kit injection. (G) Representative dot plot of PKD and conditioned mice with or without PX, showing committed progenitor populations 5 days post c-Kit injection. CLP, common lymphoid progenitors; CMP, common myeloid progenitors; GMP, granulomacrophage progenitors; LSK, Lineage^−^Sca-1^+^c-Kit^+^; LT-HSC, long-term hematopoietic stem cells; MEP, megakaryocytic and erythroid progenitors; MPP, multipotent progenitors; ST-HSC, short-term hematopoietic stem cells. Mean and SD are represented, ∗*p* < 0.05, ∗∗*p* < 0.01. (H) Analysis of the four stages in an erythroid differentiation (I, II, III, and IV) by flow cytometry in PB, spleen, and BM 5 days post c-Kit injection. I: early proerythroblasts (Ter119^med^ CD71^high^); population II: basophilic erythroblasts (Ter119^high^ CD71^high^); population III: late basophilic and polychromatophilic erythroblasts (Ter119^high^ CD71^med^); population IV: orthochromatophilic erythroblasts, reticulocytes and mature erythroid cells (Ter119^high^ CD71^low^). Mean and SD are represented. *n* = 3 non-conditioned PKD (PKD), *n* = 3 without PX (wo PX), and *n* = 2 with PX (PX). MoAb, monoclonal antibody.

We next applied our NGC regimen to a mouse model of pyruvate kinase deficiency (PKD), which is characterized by chronic hemolytic anemia. Initially, we observed that c-Kit MoAb levels in PKD mice were slightly lower than those in WT mice by day 2 (Figure S3A). To homogenize the procedure, we maintained day −5 prior to transplantation as the optimal timing for administering the anti-c-Kit antibody. Fifteen thousand WT-CD45.1-LSK^+^ cells were infused into MoAb-conditioned PKD mice, with and without PX. PX addition did not improve total engraftment or granulocyte reconstitution in PKD mice (Figures 5A and 5B), where baseline levels were significantly lower compared with WT or Rag2^−/−^ mice. However, PX significantly reduced mortality observed in the MoAb-only group (Figure 5C).

To better understand the protective role of PX in this combined regimen, we analyzed hematopoietic depletion at transplantation. PX enhanced BM depletion, particularly in Lin^−^, c-Kit^+^, MPP, ST-HSC, and LT-HSC compartments (Figures 5D and 5E). Regarding the more committed myeloerythroid progenitors, higher depletion was observed within myeloerythroid progenitor (MEP), granulocytic progenitor (GMP), and common myeloid progenitor (CMP) subpopulations, and a similar trend was observed in the common lymphoid progenitor (CLP) (Figures 5F and 5G). Despite this depletion, PX preserved erythroid progenitors, increasing the proportion of orthochromatophilic erythroblasts, reticulocytes, and more mature erythrocytes in BM without affecting PB or splenic erythroid compartments (Figure 5H). No notable changes were observed in the thymus, PB, or spleen (Figure S4A). Flow cytometry showed a reduction in CD3^+^ cells, including both CD4^+^ and CD8^+^ subtypes, but no changes in B lymphocytes (Figure S4B).

These findings suggest that PX improves the depletion of nucleated cells but has less impact on the erythroid compartment, which allows a higher survival of the NGC anemic animals.

### Increasing graft sizes and mobilization rates enhances engraftment level in PKD mice

**Figure 6:**
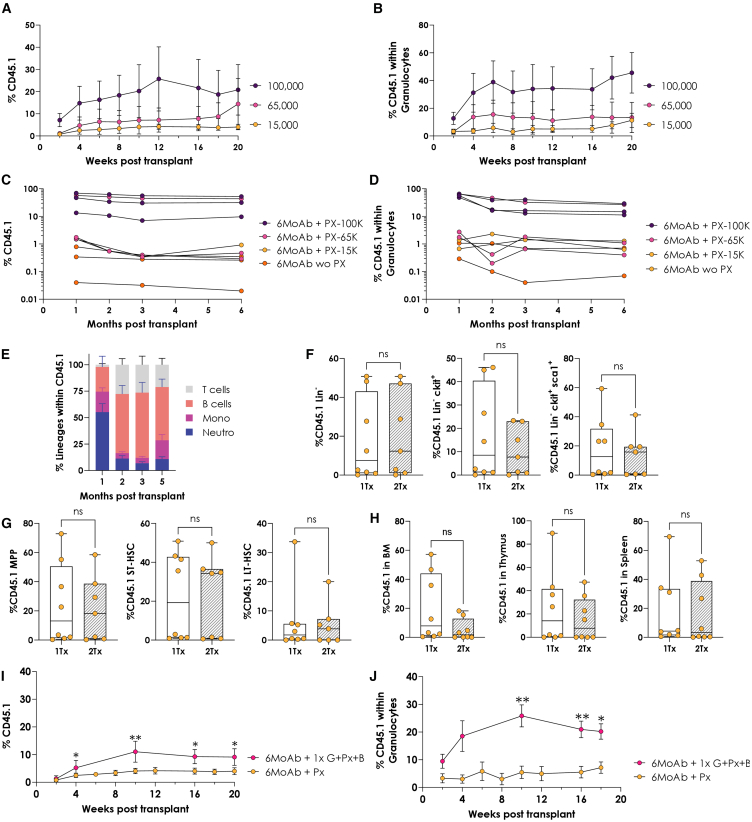
Impact of different graft dose and mobilization agents in the engraftment and multilineage reconstitution in primary and secondary recipients (A) Hematopoietic chimerism in PB of PKD mice conditioned with MoAb plus PX transplanted with different amounts of LSK^+^ cells (15,000, 65,000, and 100,000), periodically analyzed for a period of 20 weeks. (B) Granulocyte chimerism in PB of PKD mice conditioned with MoAb plus PX transplanted with different amounts of LSK^+^ cells (15,000, 65,000, and 100,000). *n* = 13 with 15,000 cells, *n* = 7 with 65,000 cells, and *n* = 7 with 100,000 cells. (C) Chimerism of CD45.1 cells peripheral blood of individual secondary animals, transplanted with 5–8 × 10^6^ million WBM from primary mice transplanted after treatment with and without PX at different cell dose. (D) Granulocytes CD45.1+ chimerism in individual secondary animals transplanted with 5–8 × 10^6^ whole bone marrow (WBM) from primary mice transplanted after treatment with and without PX at different cell dose. *n* = 3 6MoAb wo PX, *n* = 3 6MoAb with PX and 100K, *n* = 3 6MoAb with PX and 65K and *n* = 2 6MoAb with PX and 15K. (E) Lineage reconstitution within the engraftment of secondary animals. Mean and SEM are represented. T cells: CD3^+^; B cells: B220^+^; Mono: CD11b^+^Gr1^−^; Neutro: CD11b^+^Gr1^+^. (F) Comparison of CD45.1 within Lin^−^, c-Kit^+^, and LSK^+^ engraftment levels between primary recipients transplanted (1Tx-solid columns) after 6MoAb with PX and secondary recipients transplanted (2Tx-striped columns) after full irradiation conditioning. (G) Chimerism of CD45.1 cells within MPP, ST-HSC, and LT-HSC immature progenitors. (H) Chimerism of CD45.1 cells in BM, thymus, and spleen. The boxplots show the median, range, and quartiles of the data distribution. *n* = 8 from 1Tx and *n* = 7 from 2Tx. (I) Total hematopoietic chimerism in PB of PKD mice conditioned with MoAb plus PX or plus the combination of G-CSF (G), PX or Bio512 (B), (1× G + PX+B), and periodically analyzed for a period of 20 weeks. (J) Granulocyte chimerism in PB of PKD mice conditioned with MoAb plus PX or plus the combination of G-CSF (G), PX or Bio512 (B), (1× G + PX+B). *n* = 5 6MoAb with PX and *n* = 13 6MoAb with 1× G + PX+B.

To improve engraftment in PKD mice, we first increased the number of transplanted cells. Graft sizes of 15,000, 65,000, and 100,000 LSK^+^ cells/mouse were tested with the combination of MoAb plus PX. Engraftment levels improved with increasing graft size, reaching ∼21% in total PB and ∼46% in granulocytes at 20 weeks with 100,000 cells (Figures 6A and 6B).

To assess the long-term capacity of HSCs in this conditioning context, we performed secondary transplants in congenic RFP recipients, as previously described. We tracked secondary engraftment in mice conditioned with and without PX, regardless of the dose, over a 6-month period. It is important to note that due to the high mortality rate in non-PX-treated mice, we were able to analyze three animals only. Engraftment levels following secondary transplantation remained stable after a slight initial decrease during the first months with the MoAb and PX combination, while mice treated with MoAb alone exhibited near-zero levels of engraftment, both in total graft and granulocytes (Figures 6C and 6D). A successful multilineage engraftment was also observed (Figure 6E). Moreover, when we compared engraftment in MoAb + PX-treated mice across different stem cell compartments in primary and secondary recipients 6 months post-transplantation, no significant differences were observed. This stable engraftment was evident not only in the more primitive lineages (Figures 6F and 6G) but also across the total BM, spleen, and thymus (Figure 6H).

As a second approach to enhance engraftment in anemic animals, we aimed to increase the degree of mobilization. We tested granulocyte colony-stimulating factor (G-CSF),^40^ commonly used in clinical practice,^41^ and Bio5192, an antagonist of very late antigen-4 (VLA-4) that is also described as a facilitating agent to release of HSCs into the bloodstream.^32^ Both HSC mobilizers were combined with the MoAb conditioning regimen plus PX, as represented in Figure S5A. Mobilization was significantly higher and sustained compared with PX alone (Figures S5B and S5C). Based on these results, we selected the administration of pegylated G-CSF 6 days before ACK2 injection (−11 days prior to transplantation), along with a single dose of Bio5192 and PX, 1 hour before anti-c-Kit MoAb. Using this regimen, 15,000 LSK^+^ cells transplanted into MoAb-conditioned PKD mice showed significantly higher total engraftment in PB (∼9%) and more pronounced granulocyte engraftment (∼20%) (Figures 6I and 6J). These results were observed as early as 4 weeks post-transplantation and were sustained throughout the entire experimental period.

We compared the depletion efficiency of our NGC treatment (6 MoAb + G+PX+B) with lethal body irradiation, a standard genotoxic conditioning method in mice. Our NGC protocol effectively depleted bone marrow cells in a manner comparable to irradiation, but spared splenic cells, B lymphocytes, and erythroid populations in PB, spleen, and BM (Figure S6).

Our data indicate that the combination of an MoAb with PX is a feasible NGC in anemic PKD mice, which enhances graft size and/or increases mobilization rates. This protocol enables hematopoietic stem cell engraftment across all blood lineages and confirms its potential for long-term reconstitution.

### NGC regimen induces long-term and stable reversion of PKD phenotype with lower engraftment requirements compared with genotoxic regimens

**Figure 7:**
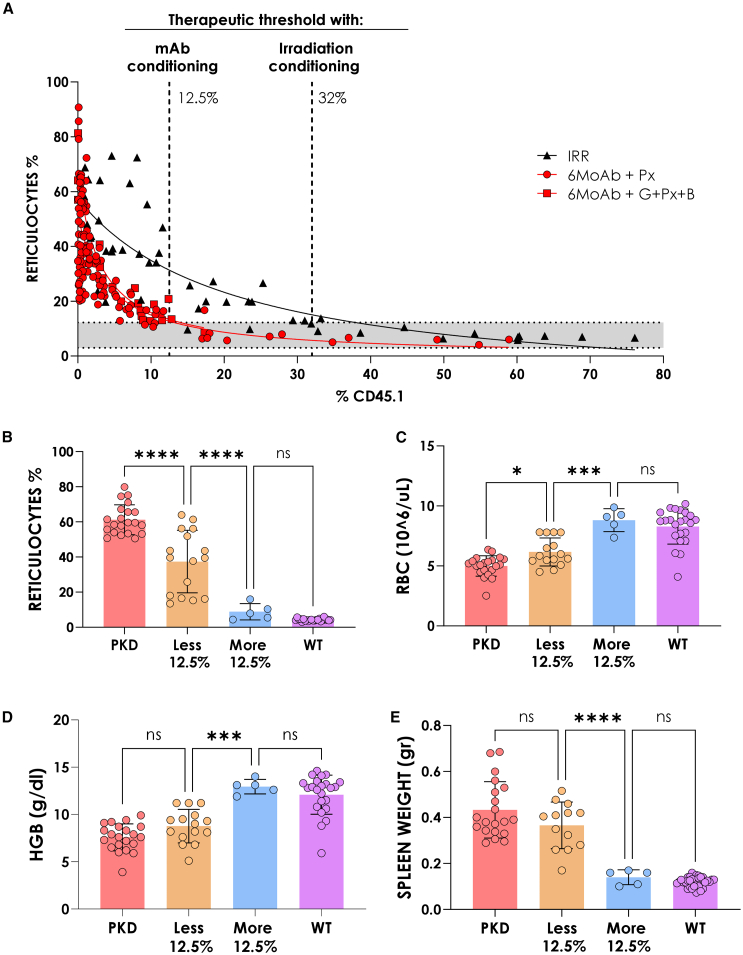
Minimum engraftment of wild-type cells required to get reversion of anemic reticulocyte phenotype and reversion of red blood cell PKD phenotype after non-genotoxic conditioning protocol 6 months post-transplant (A) Correlation between reticulocyte percentage and PB engraftment levels after non-genotoxic (red) or genotoxic (black) conditioning. Red dots (PX) and squares (PX + G + B) represent individual reticulocyte analyses and engraftment correlations following wild-type cell infusion in PKD mice conditioned with non-genotoxic regimens. Black triangles correspond to analyses from PKD mice conditioned with X-ray (genotoxic conditioning). The gray shaded area indicates the range of normal reticulocyte levels. (B) Reticulocyte percentages in PB; red bars, non-treated PKD mice; orange bars, non-genotoxic conditioned mice with and engraftment lower than 12.5%; blue bars, non-genotoxic conditioned mice with and engraftment higher than 12.5%; purple bar, wild-type animals; each dot represents the analysis of single animal; represent the mean ± SD. (C) Red blood cell (RBC) count in peripheral blood; groups as in (B). (D) Hemoglobin (HGB) quantification (f/dL); groups as in (B). (E) Spleen weight (g); groups as in (B). *n* = 50 irradiated mice; *n* = 128 conditioned mice with PX; *n* = 21 conditioned mice with G + PX+B.

Once we demonstrated improved engraftment levels and survival rates in PKD mice with the combination of MoAb plus mobilizers, we investigated whether these improvements could reverse the PKD anemia phenotype. Correlation between reticulocyte levels and engraftment of exogenous healthy cells revealed that 12.5% donor engraftment was sufficient to recover wild-type reticulocyte levels and correct anemia (Figure 7A). This therapeutic threshold was three times lower than required with conventional genotoxic conditioning (32%).^42^

We analyzed the other significant red blood cell parameters such as reticulocytes, total number of red blood cells, hemoglobin levels, and spleen weight normalized when engraftment exceeded 12.5% (Figures 7B–7E). These findings confirm that our NGC regimen enables stable and complete reversal of PKD anemic phenotype with significantly lower engraftment requirements compared with genotoxic protocols.

## Discussion

One major challenge in treating inherited hematopoietic diseases is the need for conditioning regimens to eliminate diseased HSCs and create BM niches for healthy or genetically corrected cell engraftment. Traditional genotoxic approaches, like Busulfan or total body irradiation, carry significant risks that include organ toxicity, infections, infertility, and graft-vs.-host disease (GVHD). Conditions such as SCID,^43^^,^^44^ PKD,^45^ Fanconi anemia,^46^ thalassemias,^47^^,^^48^ and sickle cell disease^49^^,^^50^ desperately need safer alternatives due to these severe complications. Although autologous transplants of genetically corrected HSPCs carry lower overall risks, there is still a critical demand for conditioning that targets HSPCs without harming other tissues.

This study demonstrates that combining monoclonal antibodies (MoAbs) with HSC mobilizers like plerixafor (PX) achieves effective HSC depletion and engraftment while reducing traditional conditioning’s toxicity. Previous studies have highlighted the efficacy of an MoAb cocktail in depleting HSCs and facilitating engraftment in mismatched donor-recipient settings.^25^ Separately, several studies have associated the use of HSC mobilizers with potential BM conditioning.^16^^,^^17^^,^^18^ We demonstrate that the combination of these strategies markedly improved engraftment reaching therapeutic thresholds. We acknowledge that patients undergoing autologous gene therapy typically require two interventions: one for HSPC mobilization and collection, and another for conditioning. Our approach replaces genotoxic conditioning with antibody-based regimens, which offer greater specificity and reduced systemic toxicity, potentially improving tolerability even following prior mobilization. Importantly, clinical experience has shown that repeated mobilization is feasible and does not impair hematopoietic recovery or graft quality.^51^^,^^52^^,^^53^^,^^54^

The MoAb cocktail targets critical pathways to achieve HSC depletion: anti-c-Kit targets a transmembrane protein essential for HSC maintenance, and anti-CD47 blocks a key anti-phagocytic signal. To further suppress host immune rejection, we included antibodies targeting CD122 (IL2Rβ), the CD40-CD40L axis, and both CD4^+^ and CD8^+^ T cells, although in a clinical setting these could potentially be replaced by approved immunosuppressive drugs. This combination achieved nearly a 3-fold increase in engraftment within both mature and immature compartments in WT mice. Although engraftment levels declined over time in secondary recipients, they remained stable thereafter, demonstrating sustained repopulating capacity. These experiments confirmed that PX synergizes with the antibody cocktail to achieve efficient HSC depletion without compromising lineage reconstitution or inducing graft failure. It is worth noting that secondary transplantation in our model involves additional biological complexity. CD45.1^+^ cells undergo two successive transplants and are repeatedly exposed to a CD45.2^+^ environment, which may impose cumulative stress and reduce their competitive fitness. In contrast, CD45.2^+^ RFP^−^ cells from the primary graft experience only one round of transplantation. This difference in transplant history and cellular stress likely contributes to the overall reduction in CD45.1^+^ engraftment observed in both groups. Despite this, the PX group maintained CD45.1^+^ cells above baseline levels, highlighting the robustness of our conditioning strategy.

Published reports on non-genotoxic antibody-based conditioning for HSPC gene therapy are limited and often focus on combining MoAbs with immunotoxins for better engraftment.^17^^,^^55^ Magenta Therapeutics developed an anti-c-Kit antibody conjugated with amanitin (eukaryotic RNA polymerase II and III), but its phase 1/2 trial (MGTA-117 - NCT05223699) was paused due to a patient’s death.^56^ However, the cause of death was unclear, and it is not known whether it was related to the therapy, as the patient had previously undergone multiple lines of treatment (5–8) and had additional co-morbidities. In contrast, our approach uses an unconjugated anti-c-Kit MoAb alongside plerixafor. While these are not yet widely used clinically in combination, both have been used in specific clinical scenarios with favorable safety profiles.^18^^,^^28^^,^^29^ Although we have not directly tested mobilization in combination with antibody-drug conjugates (ADCs), our strategy achieves comparable or superior engraftment levels without the risks associated with immunotoxins and offers a safer alternative for clinical translation. Additionally, our conditioning regimen includes anti-CD47-SIRPα alongside anti-c-Kit. Different studies have demonstrated that this combination depletes HSPCs more effectively than using anti-c-Kit alone in both murine^20^ and non-human primate^16^ models. However, recent data indicate that deglycosylated anti-CD117 antibodies do not synergize with anti-CD47 in mice.^57^ Here we used a research-grade, non-deglycosylated anti-c-Kit antibody that may explain the observed synergy and limits direct clinical translation. Importantly, the lack of synergy with one specific antibody does not rule out the potential of other clinical antibodies or formats to benefit from this approach. Notably, our results demonstrate that this combination is effective even in immunocompetent mice, suggesting that the success of co-targeting c-Kit and CD47 may depend on factors such as the antibody clone, glycosylation status, or the use of mobilization.

PX is known not only to influence HSC mobilization but also to alter the dynamics of cells in BM. In mice treated with PX alone prior to transplantation, higher engraftment was observed compared with untreated controls,^27^ which suggests that the effect was mediated by increased marrow niche availability due to the CXCR4 inhibitory effect of PX that leads to niche clearing. This is substantiated by our findings, which showed a considerably emptier BM in PX-treated animals compared with those treated only with antibodies. The cellular reorganization induced by PX could influence the overall response to conditioning, thereby enhancing treatment effectiveness.

We tested the NGC in immunodeficient (Rag-2 deficiency) and anemic (pyruvate kinase deficiency) mouse models to evaluate the therapeutic potential of this approach. In Rag2^−/−^ mice, which lack mature T and B cells but retain NK cells, we used a reduced 4-MoAb regimen excluding anti-CD4 and anti-CD8. While anti-c-Kit alone has shown efficacy in similar immunodeficient settings,^18^^,^^19^ we included anti-CD47 to mirror our other models and explore potential additive effects. Future studies will be assessed to analyze whether anti-CD47 is necessary in this context, which could help further refinement of minimal conditioning strategies for immunocompromised diseases. In Rag2^−/−^ mice, like in WT mice, the combination of mobilizers and antibodies resulted in a 2-fold increase in engraftment compared with using the MoAb alone. We also observed a more efficient recovery of B cells, T cells, monocytes, and granulocytes after the NGC procedure proposed here. Additionally, B cell recovery, a known challenge in this transplant setting,^58^ occurred earlier and was more complete with PX. Similarly, T cell recovery was accelerated, with complete thymus reconstitution achieved only in mice treated with the combined regimen. This accelerated and complete reconstitution underscores the potential of the combined conditioning regimen to support more rapid and effective immune reconstitution.

Research-grade anti-human c-Kit monoclonal antibodies have shown promising results in preclinical studies for HSCT conditioning and targeting c-Kit-expressing cancers,^59^^,^^60^ while the clinical-grade MoAb AMG191 (also known as JSP191/Briquilimab) has been evaluated in both preclinical and clinical studies. Originally developed by Amgen as AMG191, this MoAb is now being investigated in multiple clinical trials, some sponsored by Jasper Therapeutics and others as investigator-initiated studies, for conditions such as SCID,^61^ Fanconi anemia,^62^ myelodysplastic syndromes (MDSs), acute myeloid leukemia (AML),^63^ chronic granulomatous disease (CGD),^64^ and sickle cell disease (SCD).^65^ Preliminary findings from the JSP191 SCID trial indicate favorable tolerance to the drug, where 4 of 6 patients, observed beyond 24 weeks post-HSCT, exhibited successful engraftment (>5% donor granulocyte chimerism).^65^^,^^66^ However, in this study the myeloid engraftment levels in these patients appear to decrease over time, with the best outcome showing only 22% engraftment at 52 weeks. Engraftment achieved with the 4-MoAb + PX combination far exceeded levels reported here, with trends suggesting sustained engraftment over time. These findings underscore the efficacy and durability of our strategy and offer a compelling alternative to monoclonal antibody-only regimens for primary immunodeficiencies (PIDs).

In PKD mice, faster clearance of anti-c-Kit was observed compared with WT mice (1 day shorter),^13^^,^^37^ likely due to the splenomegaly^67^ and elevated erythropoietin (EPO) levels.^68^ Stress erythropoiesis may also contribute, with progenitors preferentially homing to the spleen and redistributing engraftment away from the BM.^69^ This effect, along with the high expression of CD47 during stress-induced erythropoiesis,^70^ may accelerate antibody activity and further decrease engraftment in PKD mice. However, PX mitigated these effects by mobilizing progenitors into the bloodstream, where they are effectively targeted by anti-c-Kit. In contrast, mature erythroid cells, which lack c-Kit, are spared. These findings highlight how mobilization can influence antibody targeting and suggest that optimizing cell distribution may improve conditioning selectivity.

We show that the combination of MoAbs plus PX significantly reduced mortality in PKD mice compared with MoAbs alone or conventional irradiation. In contrast to the aggressive, genotoxic conditioning methods that cause substantial damage to the BM niche, our non-genotoxic approach selectively targets HSPCs. PX further helps this selectivity by mobilizing HSPCs, which reduces the need for antibodies to penetrate the BM. This BM niche preservation can affect the effectiveness of the treatment. In fact, while conventional conditioning methods require about 30% healthy cell engraftment to reverse the PKD phenotype, our strategy using MoAbs plus PX achieves therapeutic outcomes with just 12.5% engraftment. This reduced threshold has critical implications for gene therapy and gene editing strategies, where achieving high levels of corrected cell engraftment can be challenging.

In summary, our study establishes a completely non-genotoxic conditioning regimen combining antibodies and mobilizers for effective HSPC engraftment. This method achieves high-level long-term engraftment of healthy donor HSPCs and ensures sustained phenotypic correction in both primary immunodeficiencies and congenital anemias. These findings provide substantial evidence supporting the potential translation of this non-genotoxic approach to HSCT conditioning to the clinic.

## Materials and methods

### Experimental animals

Five mouse strains were used. C57BL/6J (B6) mice were obtained from the Jackson Laboratory (Bar Harbor, ME) and C57BL/6N-Rag2Tm1/CipheRj (Rag2^−/−^) mice were obtained from Janvier (Le Genest-Saint-Isle, France). AcB55 recombinant mice (also recorded as PKD mice) carrying the 269T>A loss-of-function mutation in the *pklr* gene were obtained from Emerillon Therapeutics (Montreal, Quebec, Canada). As the genetic background of ACB55 was both 87.5% A/J + 12.5% C57BL/6J, we backcrossed onto an inbred mouse strain B6 for N7 generations. Congenic donor mice CD45.1+ (B6.SJL-Ptprca Pepcb/BoyJ) were generated in the B6SJL-PtprcaPep3b/BoyJ strain (Pep3B; Ly5.1 phenotype), congenic with C57BL/6J (B6; Ly5.2) mice. RFP C57BL/6 mice were kindly provided by Dr. David A. Brenner (UCSD, CA). All experimental procedures were carried out according to Spanish and European regulations at the CIEMAT animal facility (registration number 28079-21 A). Mice used in the experiment were 6–12 weeks old (male and female), age-matched, and randomized to experimental groups.

### Clearance of c-Kit antibody

Mice were intravenously injected with 500 μg of c-Kit antibody and blood samples were collected at 2, 4, and 5 days after the injection for analysis. Blood was centrifuged for 10 min at 1,400 rpm to extract plasma. Plasma was diluted 1:10 and incubated with BDTM CompBeads Anti-Rat and Anti-Hamster Ig,k (Cat:51-90-9000949, BD Biosciences) 20 min at 4°C in darkness. Then the beads were washed and stained with 1 μL/50 μL of a secondary antibody (Goat F(ab')2 Anti-Rat IgG (H + L)-TRIC, Catalog: R40106). Thereafter, beads were washed with PBS and centrifuged for 7 min at 1,400 rpm at room temperature. After all, beads were resuspended in PBS with DAPI and analyzed by flow cytometry.

### Non-genotoxic conditioning treatment

Mice were conditioned with a combination of six different MoAbs over 7 days prior to transplantation following a protocol modified from George et al.^25^ For anti-CD47 (clone mIAP410), mice received 100 μg on day −7, followed by 500 μg daily from day −5 through day −2. For anti-c-Kit (clone ACK2), a 500-μg intravenous injection was given on day −5. Fifteen minutes prior to anti-c-Kit injections, mice received 400 μg of diphenhydramine intraperitoneally. Both anti-CD4 (clone GK1.5) and anti-CD8 (clone YTS169.4) were given as 100-μg injections daily from day −2 through day 0. For anti-CD122 (clone Tm-β1), 250 μg was given on day −2. For anti-CD40L (clone MR-1), 500 μg was given on day 0. Day 0 corresponded to the day of transplantation. All MoAbs were obtained from BioXCell (Lebanon, NH, USA). Mobilized mice received a single dose of plerixafor (Mozobil 5 mg/kg) subcutaneously on day −5 one hour before anti-c-Kit injection alone or combined with subcutaneously pegylated G-CSF 6 days before the anti-c-Kit injection (−11 days prior to transplantation), and/or one dose of Bio5192 subcutaneously (−5 days prior to transplantation) (Figure S1).

### Transplantation protocols

BM-derived hematopoietic stem cells with an LSK (Lin^−^Sca1^+^c-Kit^+^) phenotype were isolated from femora and tibiae by flushing. BM cells were stained for LSK markers using MoAbs listed on Table S1 and sorted via fluorescence-activated cell sorting (FACS) using a BD Influx (BD Biosciences, Franklin Lakes, NJ, USA).

For primary transplantation, 15,000 LSK^+^ cells of BM from the B6.SJL-*Ptprc*a/b*Pep*3b/BoyJ(P3B) (Ly5.1^+^) mice were transplanted into C57Bl6 WT or Rag2^−/−^ mice, while between 15,000 and 100,000 LSK^+^ cells were transplanted in PKD recipients (males and females). Five to 6 months post-transplantation, primary recipients were euthanized, and BM cells collected from femora and tibiae and analyzed for population content. For secondary transplantation, 5 × 10^6^ to 8 × 10^6^ BM cells were intravenously infused into myeloablated RFP C57BL/6 mice that received two doses of 4.5 Gy, spaced 24 h apart with X-ray equipment MG324 (300 kV, 12.8 mA, Philips, Hamburg, Germany).

### Sample extraction

At the final stage of the conditioning treatment and throughout the follow-up period, samples were collected from treated and control mice for analysis, including PB, BM, spleen, thymus, and liver. PB samples were routinely obtained by collecting a maximum of 200 μL through lateral tail vein bleeding (Microvette, Sarstedt, Nümbrecht, Germany) or via cardiac puncture under CO_2_ anesthesia at the final stage. Spleen, thymus, and BM were surgically removed and placed in PBS. Spleens were measured and weighed. To prepare spleen and thymus samples for analysis, organs were mechanically disaggregated using a swab and filtered through a 40-μm nylon filter (BD/Becton, Dickinson and Company, New Jersey, USA). All samples were preserved for flow cytometry and hematological counts.

### Hematological counts and flow cytometry antibodies

PB hematological counts were analyzed using a Sysmex XN-1000 analyzer (Sysmex, Kobe, Japan). Mouse engraftment and mobilization kinetics in different lineage compartments were analyzed by flow cytometry using specific antibodies (Tables S2, S3, S4, and S5) and washed with flow cytometry buffer (PBS with 0.5% BSA and 0.05% sodium azide). When needed, erythrocytes were lysed in ammonium chloride lysis solution (0.155 mmol/L NH4Cl + 0.01 mmol/L KHCO3 + 10−4 mmol/L EDTA) before antibody staining. Acquisition was performed on the LSRFortessa cell analyzer (BD Biosciences). DAPI or PI was used as a viability marker. Offline analysis was conducted with FlowJo Software.

### Statistical analysis

Statistical analysis was performed using GraphPad Prism version v9.4.1 (GraphPad Software, San Diego CA, USA). Normal distribution was assessed using the Shapiro-Wilk test. Student’s t test or Mann-Whitney *U* tests were used based on the normality of data to compare two groups. For more than two groups, one-way ANOVA with Tukey’s multiple comparison tests was used for normally distributed data, while Kruskal-Wallis test with Dunn’s multiple comparison test was employed for nonparametric analyses. Results are presented as mean ± standard error of the mean (SEM) or in a boxplot showing the median and the interquartile range. The significance is expressed as *p* < 0.0001(∗∗∗∗), *p* < 0.001(∗∗∗), *p* < 0.01(∗∗), or *p* < 0.05(∗).

## Data availability

All data supporting the findings of this study are available from the corresponding authors upon request.

## Acknowledgments

The authors would like to thank Mrs. Aurora de la Cal, María del Carmen Sánchez, Soledad Moreno, Nadia Abu-Sabha, Montserrat Aldea, Maria Jesús Arias, Eveyanira Carolina Piñero, and Sergio Losada for their dedicated administrative help; Norman Feltz for his kind revision of the language manuscript; and Dr. Julian Sevilla and Dr. Guzmán López de Hontanar Torres for their helpful clinical information and discussions. The authors also thank Fundación Botín for promoting translational research at the Division of Hematopoietic Innovative Therapies of the CIEMAT. CIBERER is an initiative of the “Instituto de Salud Carlos III” and “Fondo Europeo de Desarrollo Regional (FEDER)” and Instituto de Investigación Sanitaria Fundación Jiménez Díaz. This work was supported by grants from “Ministerio de Economía, Comercio y Competitividad y Fondo Europeo de Desarrollo Regional (FEDER)” (SAF2017-84248-P), “Ministerio de Ciencia e Innovación” (PID2020-119637RB-I00), “Instituto de Salud Carlos III (ISCIII)/Red Española de Terapias Avanzadas RICORS/TERAV” (RD21/0017/0027, supported by the European Union—NextGenerationEU, Plan de Recuperación Transformación y Resiliencia), “Fondo de Investigaciones Sanitarias, Instituto de Salud Carlos III” (Red TERCEL; RD16/0011/0011), Comunidad de Madrid (AvanCell, B2017/BMD-3692), “Proyectos generación de conocimientos 2023” (PID2023-152564OB-I00), and CIBERER.

## Author contributions

I.O.-P. and R.S.-D. designed and performed the experiments and wrote the manuscript; O.A.-F., A.G.-T., and M.L.-S. helped with the experimental procedures; J.-C.S. designed the experiments, wrote the manuscript, and provided grant support.

## Declaration of interests

J.-C.S. is a consultant of Rocket Pharmaceuticals and DanausGT Biotechnology and holds shares from Rocket Pharmaceuticals. M.L.-S. is a co-founder of Kiji Therapeutics.

## Supplemental information

Document S1. Figures S1–S6 and Tables S1–S5

Document S2. Article plus supplemental information
