# Supplementary material for: HSC engraftment is enhanced by combining mobilization with anti-C-Kit and Anti-CD47-based conditioning in hematopoietic transplant
Source: Mol Ther. 2025 Jul 16;33(10):5044–60. doi: 10.1016/j.ymthe.2025.07.012 (PMC12848187; doi:10.1016/j.ymthe.2025.07.012)
Supplement: Document S1. Figures S1–S6 and Tables S1–S5 [file mmc1.pdf]

YMTHE, Volume *33*

Supplemental Information

HSC engraftment is enhanced by combining mobilization with anti-C-Kit and Anti-CD47-based conditioning in hematopoietic transplant

Isabel Ojeda-Perez, Omaira Alberquilla-Fernandez, Aida García-Torralba, Mercedes Lopez-Santalla, Rebeca Sánchez-Domínguez, and Jose-Carlos Segovia

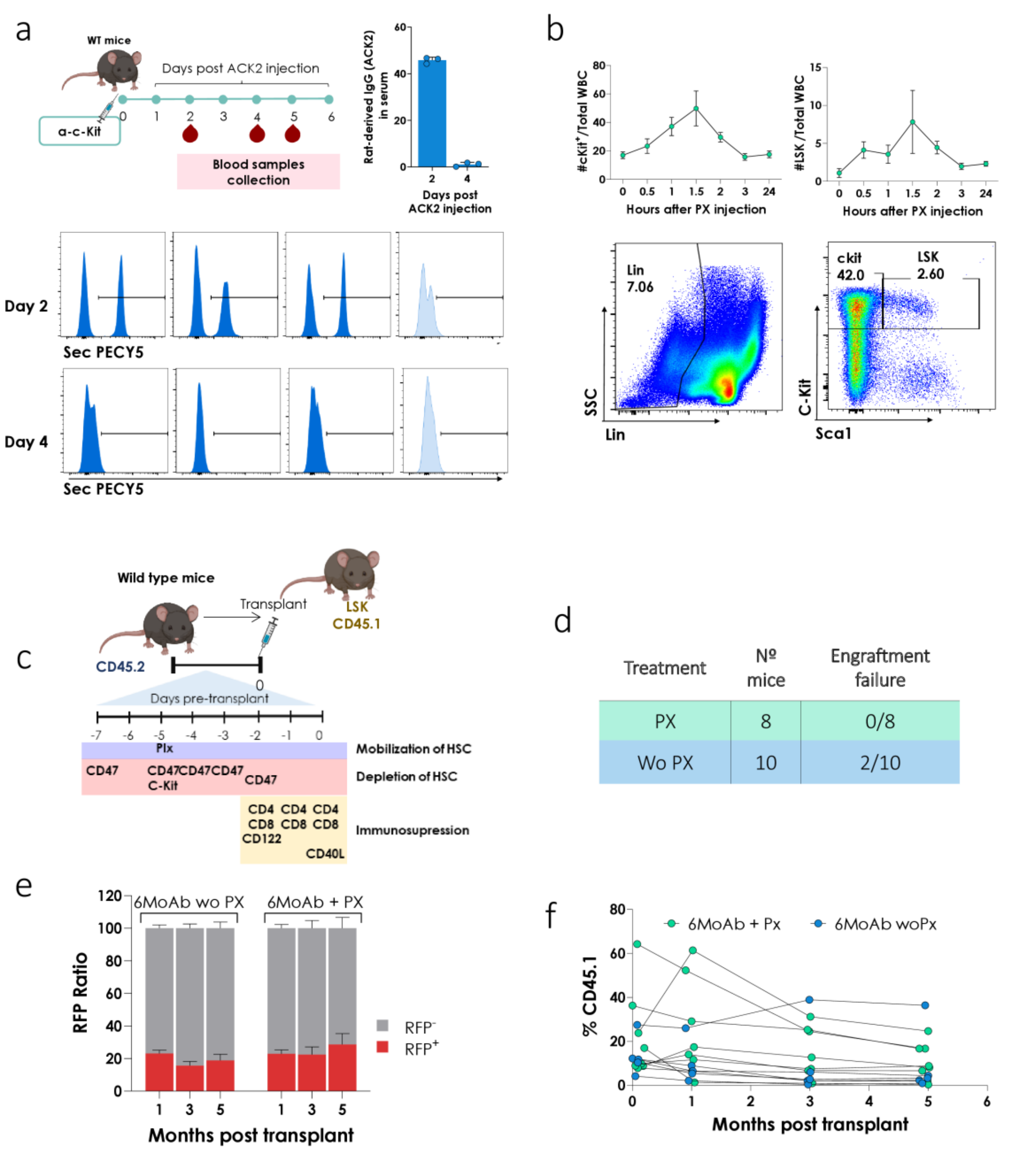

Figure S1. Establishment of the conditioning protocol.

a. Top: Scheme of c-kit injection and days of blood samples collection and quantification of the percentage of ACK2 IgG in the serum of mice 2 and 4 days after ACK2 injection. Mean and SD are represented. Bottom: Clearance of anti-c-kit antibody for WT mice. Each dot plot represents an individual mouse. Dark colours correspond to the mice that received ACK2 injection, while light colours represent the control samples without ACK2 injection, incubated with the secondary antibody. **b**. Mobilization kinetics of stem cells populations in PB of WT mice after Px injection. Top: Changes in c-Kit and LSK populations relative to WBC in PB. Bottom: Representative dot plot from the cytometry analysis of the aforementioned populations. N=10 at 0 and 24 hours. The animals were divided into two groups of N=5 for the remaining time points. Mice were bled immediately before Px injection (baseline) and subsequently at 30, 60, 90, 120 and 180 minutes post-injection, with a final sample collected at 24 hours. **c**. Scheme of non-genotoxic conditioning

regimen with the use of 6mAb in PKD or WT mice. Non-genotoxic conditioning was performed prior to transplantation on day 0. Mice received anti-CD47 on day -7, followed by daily injections from day -5 through day -2. Anti-c-kit was administered intravenously on day -5. Both anti-CD4 and anti-CD8 were given via daily injections from day -2 through day 0. Anti-CD122 was injected on day -2 and anti-CD40L on day 0. **d**. Engraftment failure in mice conditioned with or without the combination of plerixafor and monoclonal antibodies. **e**. Proportion of RFP cells in secondary recipients in both conditions. Data are shown as RFP ratio at 1, 3, and 5 months post-transplant. **f.** Analysis of donor chimerism (%CD45.1) in peripheral blood from individual transplanted mice treated with 6MoAb plus plerixafor (6MoAb + PX) or 6MoAb alone (6MoAb wo PX).

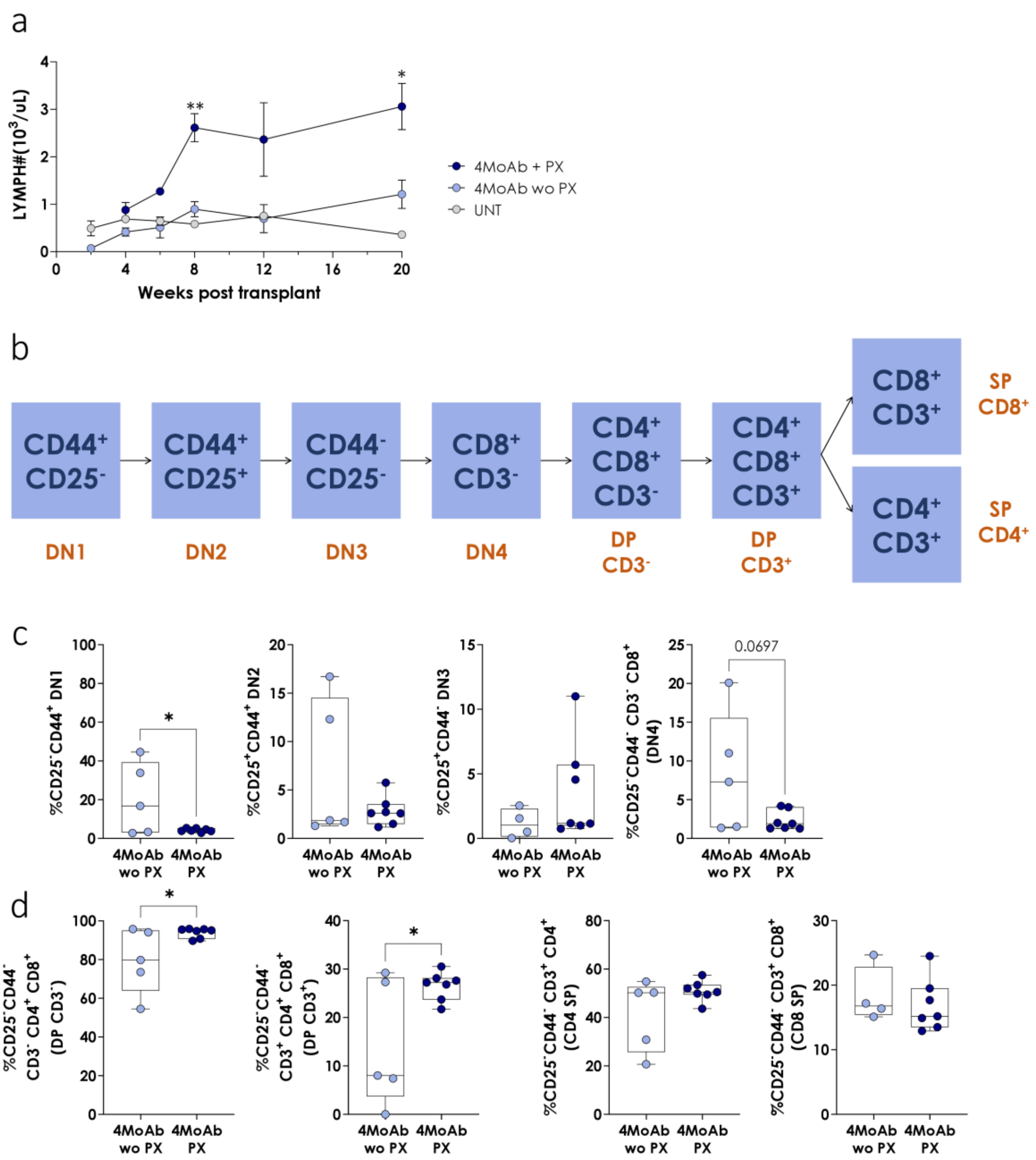

Figure S2. Impact of NGC treatment with and without Px on thymic recovery.

a. Values of absolutes lymphocytes (LYMPH#, $\times 10^3/\mu L$) measured by hematology analyzer at different time points post-transplantation. **b**. Schematic representation of the markers and nomenclature for thymic differentiation stages, as defined in [1,2]. Populations include DN1 ($CD44^+CD25^-$), DN2 ($CD44^+CD25^+$), DN3 ($CD44^-CD25^+$), DN4 ($CD44^-CD25^-CD3^-CD8^+$), double-positive (DP) $CD3^-$ ($CD44^-CD25^-CD3^-CD4^+CD8^+$), DP $CD3^+$ ($CD44^-CD25^-CD3^+CD4^+CD8^+$), single-positive (SP) CD4 ($CD44^-CD25^-CD3^+CD4^+$), and SP CD8 ($CD44^-CD25^-CD3^+CD8^+$). **c**. Percentages of DN populations (DN1, DN2, DN3, DN4) with and without Px treatment. **d**. Percentages of differentiated thymic populations, including DP $CD3^+$, SP CD4, and SP CD8, in mice treated with and without Px.

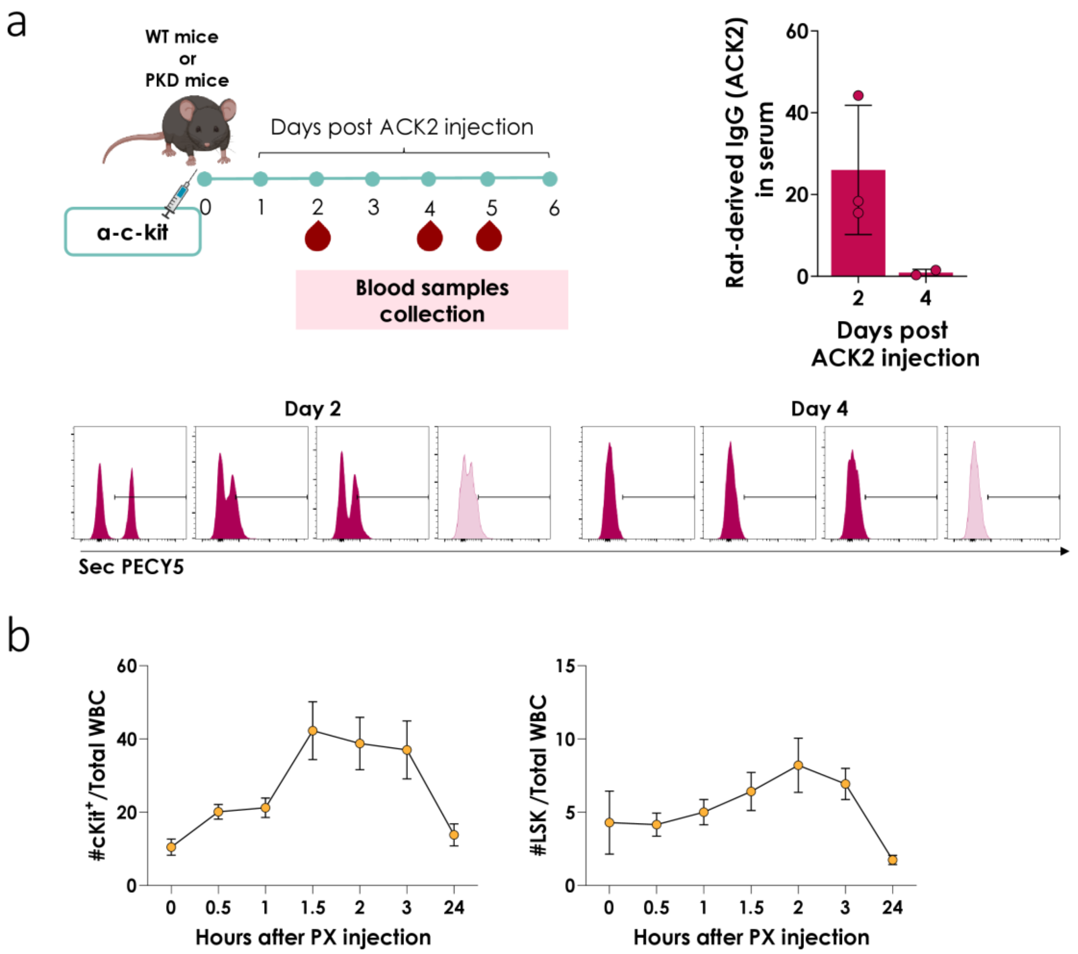

Figure S3. Establishment of the conditioning protocol in PKD mice.

a. Top: Scheme of c-kit injection and days of blood samples collection and quantification of the percentage of ACK2 IgG in the serum of mice 2 and 4 days after ACK2 injection. Mean and SD are represented. Bottom: Clearance of anti-c-kit antibody for PKD mice. Each dot plot represents an individual mouse. Dark colours correspond to the mice that received ACK2 injection, while light colours represent the control samples without ACK2 injection, incubated with the secondary antibody. **b**. Mobilization kinetics of stem cells populations in PB of PKD mice after Px injection. Changes in c-Kit and LSK populations relative to WBC in PB. N=10 at 0 and 24 hours. The animals were divided into two groups of N=5 for the remaining time points. Mice were bled immediately before Px injection (baseline) and subsequently at 30, 60, 90, 120 and 180 minutes post-injection, with a final sample collected at 24 hours.

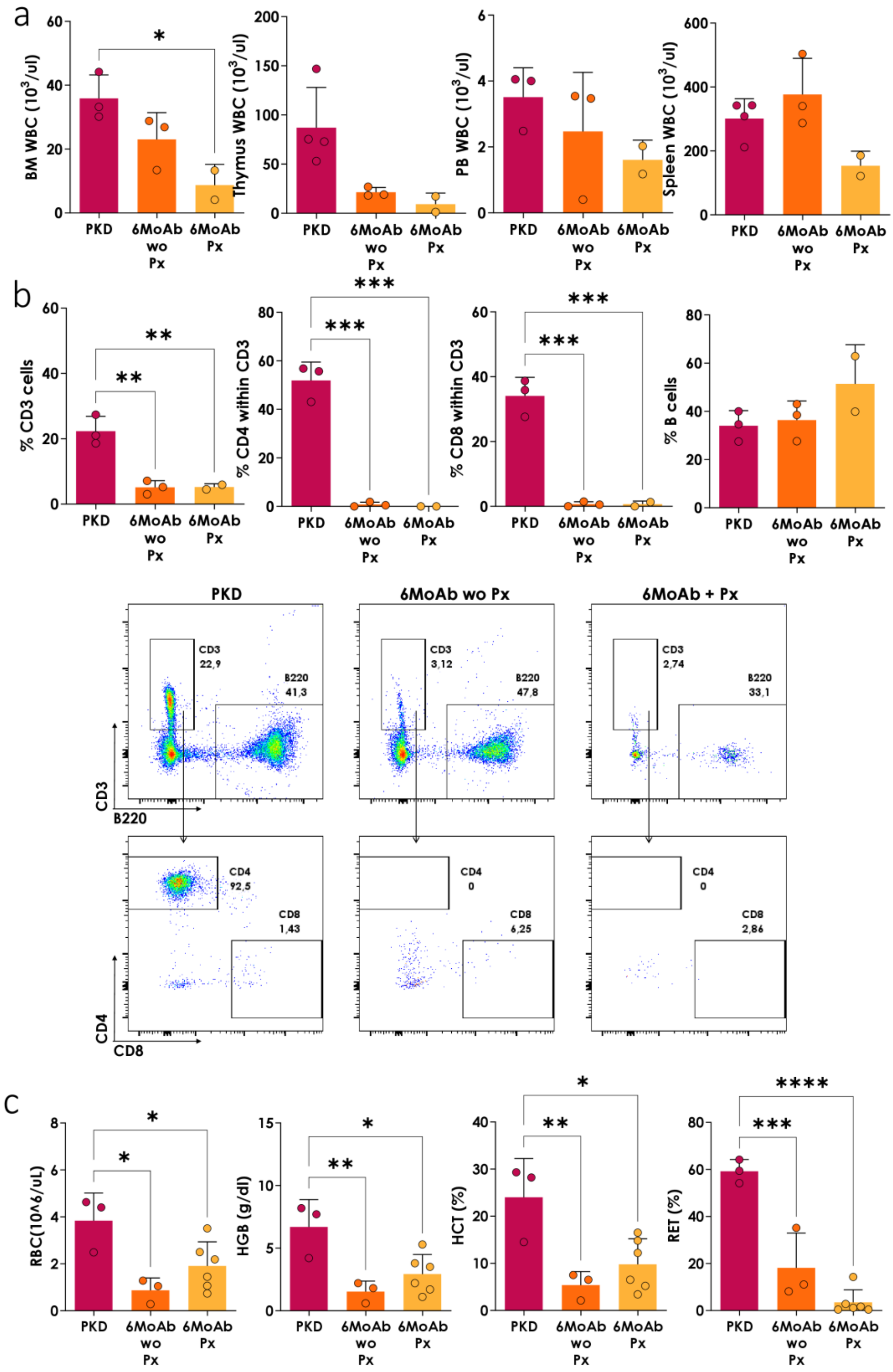

Figure S4.White blood cells parameters in spleen thymus and peripheral blood with or without Px injection.

a. Cell count of WBC (expressed as 10^3/µl) in hematopoietic organs, such as BM, thymus, PB and Spleen at the time of the sacrifice. **b**. Flow cytometry studies of lymphoid subsets

in PB. Mean and SD are represented. CD3: CD11b$^-$ B220$^-$ CD3$^+$; CD4: CD11b$^-$ B220$^-$ CD3$^+$CD4$^+$ CD8$^-$; CD8: CD11b$^-$ B220$^-$ CD3$^+$CD4$^-$ CD8$^+$; B cells: CD11b$^-$ B220$^+$ CD3$^+$. **c**. Red blood cell parameters after conditioning in PB. RBC: Red blood cells; HGB: Hemoglobin; HCT: Hematocrit; RET: Reticulocytes. Mean and SD are represented.

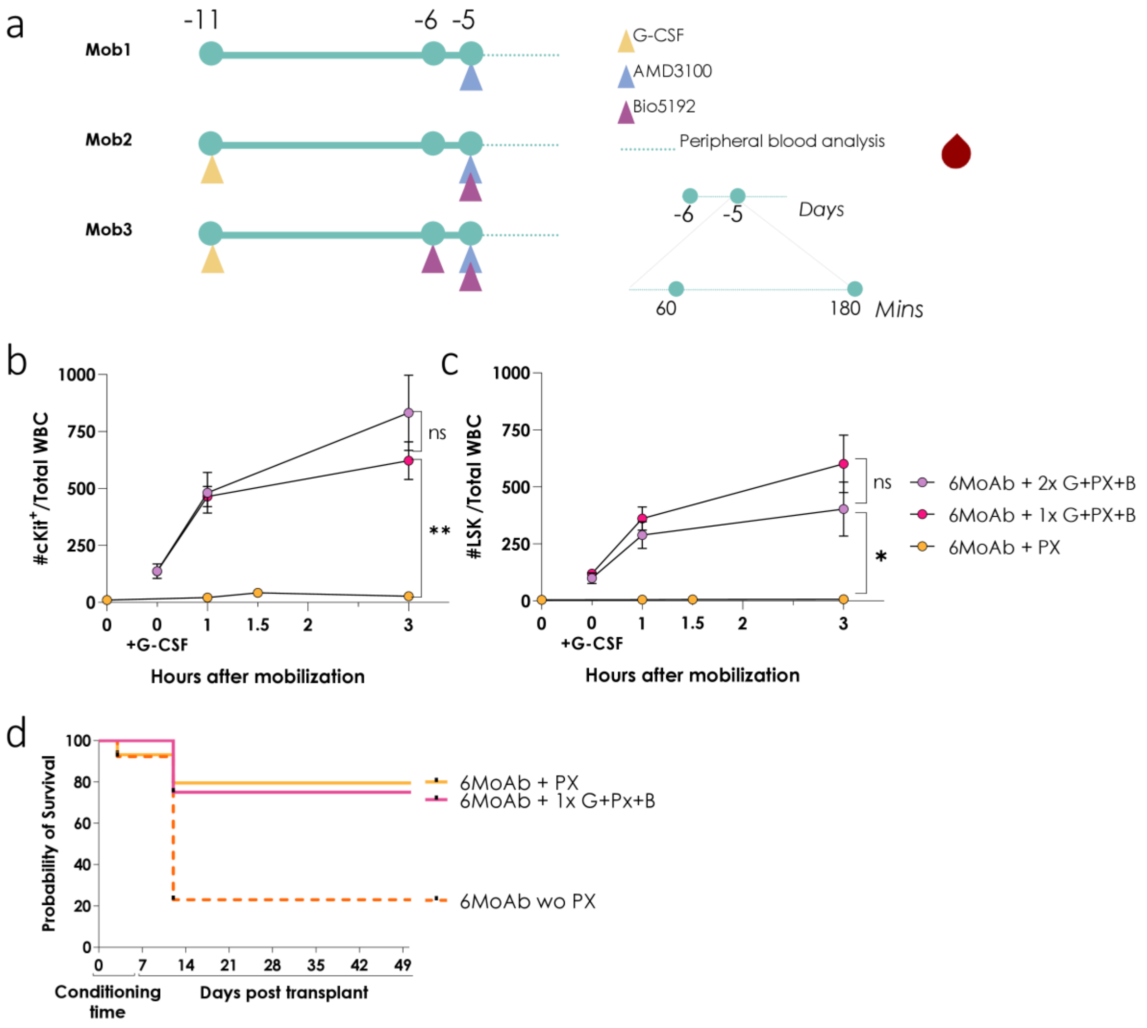

Figure S5. HSPC mobilization studies with new mobilizer agents.

a. Scheme of different mobilization protocols tested and timing of analysis in PKD mice. Px: Received only Plerixafor one hour before ACK2 (anti-c-Kit) injection; 1xG+Px+B: Pegylated G-CSF was administered six days before ACK2 injection (-11 days before transplantation), along with a single dose of Bio5192. Px was administered only once, coinciding with the Bio5192 dose, one hour before ACK2; 2xG+Px+B: Similar to the previous regimen, but Bio5192 was administered in two doses, one the day before and another one hour before ACK2. **b**. Absolute numbers of c-kit populations at different time points during the mobilization protocols. **c**. Absolute numbers of LSK populations at different time points during the mobilization protocols. **d**. Survival rates of PKD mice who underwent a conditioning treatment with the antibody cocktail, without Px, with Px or with the combination of G+Px+B.

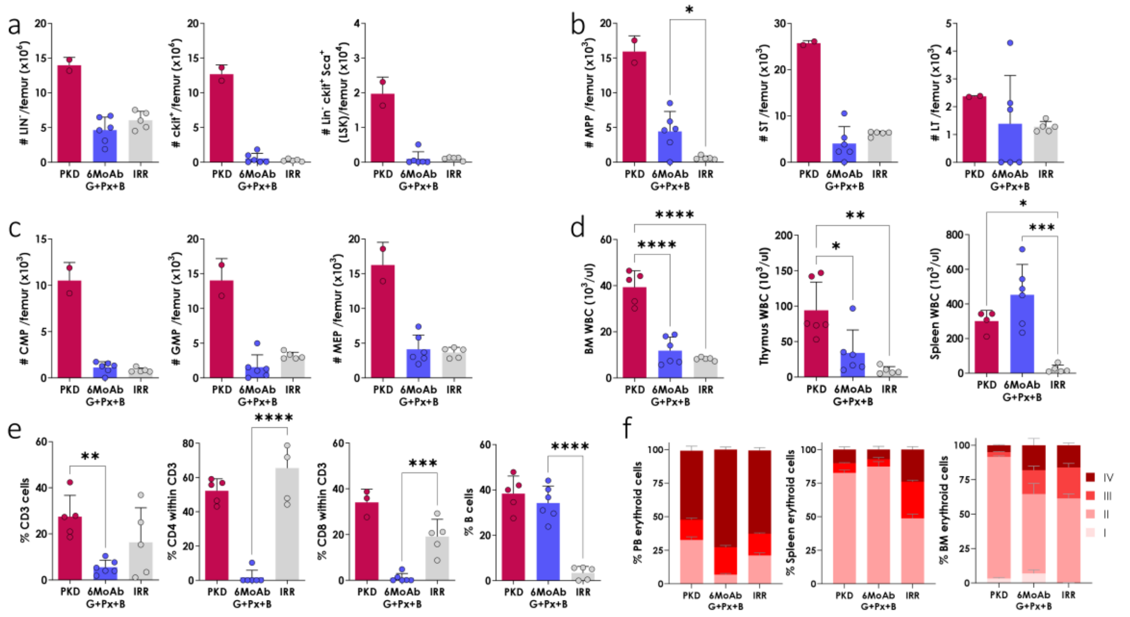

Figure S6. Comparison of BM depletion after treatment with 6MoAb cocktail plus mobilizer cocktail conditioning and irradiation.

a. Total number per femur of non-mature cells (Lin$^-$) and committed progenitors (Lin$^-$c-kit$^+$ and Lin$^-$c-kit$^+$ Sca-1$^+$) in the BM of the animals treated with the 6mAb cocktail with mobilizer cocktail (G+ Px+ B) or with irradiation (IRR). Non-treated PKD animals (PKD) were also analyzed as controls. **b**. Variations in immature phenotypes, such as MPP: LSK$^+$ CD150$^-$ CD48$^+$; ST: LSK$^+$ CD150$^-$ CD48$^-$; LT: LSK$^+$ CD150$^+$ CD48$^-$. **c**. Total number per femur of lineage committed progenitors including CMP: LSK$^+$CD34$^+$FCRɣ$^-$; GMP: LSK$^+$CD34$^+$ FCRɣ$^+$; MEP: LSK$^+$ CD34$^-$ FCRG$^-$. LSK, Lineage$^-$Sca-1$^+$c-Kit$^+$; MPP, multipotent progenitors; ST-HSC, short-term hematopoietic stem cells; LT-HSC, Long-term hematopoietic stem cells; CLP, common lymphoid progenitors; CMP, common myeloid progenitors; GMP, granulomacrophage progenitors; MEP, Megakaryocytic and erythroid progenitors. **d**. Cell count of WBC (expressed as 10^3/µl) in hematopoietic organs, such as BM, thymus, PB and Spleen at the time of the sacrifice. **e**. Flow cytometry studies of lymphoid subsets from PB. Mean and SD are represented. CD3: CD11b$^-$ B220$^-$ CD3$^+$; CD4: CD11b$^-$ B220$^-$ CD3$^+$CD4$^+$ CD8$^-$; CD8: CD11b$^-$ B220$^-$ CD3$^+$CD4$^-$ CD8$^+$; B cells: CD11b$^-$ B220$^+$ CD3$^-$. **f**. Analysis of erythroid compartments by flow cytometry in PB, spleen and BM . We analysed the four stages in an erythroid differentiation (I, II, III and IV). I: early proerythroblasts (Ter119^{med} CD71^{high}); population II: basophilic erythroblasts (Ter119^{high} CD71^{high}); population III: late basophilic and polychromatophilic erythroblasts (Ter119^{high} CD71^{med}); population IV: orthochromatophilic erythroblasts, reticulocytes and mature erythroid cells (Ter119^{high} CD71^{low}). Mean and SD are represented, $^*p<0.05$, $^{**}p<0.01$.

Table S1. Murine monoclonal antibodies for LSK^{+} (Lin^{-} Sca-1^{+} c-kit^{+}) cell sorting and analyses.

Antibody	Conjugated Fluorochrome	Clone	Antibody Concentration (µg /10^{6} cells)	Cells Marker	Vendor
**Anti-CD3**	FITC	145-2C11	0.15	T lymphocytes	BD Pharmingen
**Anti-GR1** (Ly6G/6C)		RB6-8C5	0.15	Granulocytes	BioLegend
**Anti-CD11b** (Mac1)		M1/70	0.3	Myeloid	eBioscience
**Anti-B220** (CD45R)		RA3-6B2	0.15	B lymphocytes	BioLegend
**Anti-Ter119**		TER119	0.15	Mature erythrocytes and progenitors	BioLegend
**Anti-SCA-1** (Ly-6A/E)	PE	E13-161.7	0.06	Hematopoietic progenitors	BD Pharmingen
**Anti-c-kit** (CD117)	PECy7	2B8	0.06		BioLegend

Abbreviations: FITC, fluorescein isotiocyanate; PE, phycoerythrin

Table S2. Monoclonal antibodies used for mobilizations analysis.

Epitope	Fluorochrome	Clone	Provider	Catalog	Vol/50µl
CD3	PECY5	145-2C11	BD Pharmingen	553065	0.16µl
CD45R/B220	PEFIRE700	RA3-6B2	BioLegend	103280	0.33µl
Ly-6A/E (Sca 1)	APC-Cy7	D7	BioLegend	108126	0.33µl
Ly-6G	BV421	1A8	BD Pharmingen	561104	0.33µl
Ly-6C	PECY7	HK1,4	BioLegend	128017	0.33µl
CD11b	A647	M1/70	BioLegend	101218	0.5µl
CD16/32	PercP-eFluor710	93	eBioscience	46-0161-82	0.16µl
CD34	FITC	RAM34	eBioscience	11-0341-81	0.5µl
CD71	BV510	RI7217	BioLegend	113823	0.5µl
CD117(c-kit)	BV711	2B8	BioLegend	105835	0.33µl
TER-119	A700	TER119	BioLegend	116220	0.33µl
CD135	PE	A2F10,1	BD Pharmingen	553842	0.33µl
F4/80 Antigen	BV650	BM8	BioLegend	123149	0.33µl

Abreviations: PE, phycoerythrin; BV711, Brilliant violet 711; FITC, fluorescein isotiocyanate; Pecy5; PE-cyanine5; APCCy7, APC-Cyanine7; A647, AlexaFluor 647; A700, AlexaFluor 700; BV421, Brilliant violet 421; BV510, Brilliant violet 510; BV650, Brilliant violet 650; PercP-eFluor710; Pecy7; PE-cyanine7

Table S3. Monoclonal antibodies employed for the analysis of mouse engraftment.

	Epitope	Fluorochrome	Clone	Provider	Catalog	Vol/50µl
1º transplant	mCD45.2	APC	104	BioLegend	109814	1µl
	mCD45.1	PE	A20	BioLegend	110708	1µl
2º transplant	mCD45.2	APC	104	BioLegend	109814	1µl
	mCD45.1	BV711	A20	BioLegend	110739	1µl

Abbreviations: APC, allophycocyanin; PE, phycoerythrin; BV711, Brilliant violet 711

Table S4. Monoclonal antibodies employed for the analysis of linages reconstitution.

	Epitope	Fluorochrome	Clone	Provider	Catalog	Vol/50µl
			Linages analysis			
1º Tx	mCD45.1	PE	A20	BioLegend	110708	1µl
	CD11b	BV711	M1/71	BioLegend	101242	1µl
	Ly-6G/6C	FITC	RB6-8C5	BioLegend	108406	1.5µl
	CD45R/B220	PECy5	RA3-6B2	BD Pharmingen	553091	0.5µl
	CD3	APCCy7	17 A2	BioLegend	100222	1µl
2º Tx	mCD45.1	BV711	A20	BioLegend	110739	1µl
	CD11b	A647	M1/70	BioLegend	101218	1µl
	Ly-6G/6C	FITC	RB6-8C5	BioLegend	108406	1.5µl
	CD45R/B220	PEFire 700	RA3-6B2	BioLegend	103280	0.5µl
	CD3	APCCy7	17 A2	BioLegend	100222	1µl
			Erythoid analysis			
1º Tx	mCD45.1	PE	A20	BioLegend	110708	1µl
	Ter119	A700	TER119	BioLegend	116220	1.5µl
	CD71	FITC	C2	BD Pharmingen	553266	2.5µl
2º Tx	mCD45.1	BV711	A20	BioLegend	110739	1µl
	Ter119	A700	TER119	BioLegend	116220	1.5µl
	CD71	FITC	C2	BD Pharmingen	553266	2.5µl

Abbreviations: 1º Tx: primary transplant; 2º Tx: secondary transplant. PE, phycoerythrin; BV711, Brilliant violet 71; FITC, fluorescein isotiocyanate; Pecy5; PE-cyanine5; APCCy7, APC-Cyanine7; A647, AlexaFluor 647; A700, AlexaFluor 700.

Table S5. Monoclonal antibodies employed for the analysis of long term populations.

	Epitope	Fluorochrome	Clone	Provider	Catalog	Vol/50µl
1º Tx	mCD45.1	PE	A20	BioLegend	110708	1µl
2º Tx	mCD45.1	PerCPCy5.5	A21	BioLegend	110727	1µl
common for 1º and 2º Tx	CD3	FITC	145-2C11	BD Pharmingen	553062	1µl
	CD11b		M1/70	eBioscience	11-0112-85	1µl
	Ly-6G/6C		RB6-8C5	BioLegend	108406	1µl
	CD45R/B220		RA3-6B2	BioLegend	103206	1µl
	Ter119		TER119	BioLegend	116206	1µl
	Ly-6A/E (Sca-1)	APCCy7	D7	BioLegend	108126	1µl
	CD117 (c-kit)	BV711	2B8	BioLegend	105835	1µl
	CD48	PECy7	HM48-1	BD Pharmingen	560731	1µl
	CD150	APC	TC15-12F12.2	BioLegend	115910	1.5µl

Abbreviations: 1º Tx: primary transplant; 2º Tx: secondary transplant . PE, phycoerythrin; BV711, Brilliant violet 71; FITC, fluorescein isotiocyanate; Pecy7; PE-cyanine7; APCCy7, APC-Cyanine7; : APC, allophycocyanin.
